# Reliability of a Modified 24 h Dietary Recall and Veggie Meter to Assess Fruit and Vegetable Intake in New Zealand Children

**DOI:** 10.3390/nu17203293

**Published:** 2025-10-20

**Authors:** Varshika V. Patel, Thalagalage Shalika Harshani Perera, Elaine Rush, Sarah McArley, Carol Wham, David S. Rowlands

**Affiliations:** 1 School of Sport, Exercise and Nutrition, Massey University, Auckland 0632, New Zealand; varshika.vpatel@gmail.com (V.V.P.); shalika.harshani@gmail.com (T.S.H.P.); s.m.mcarley@massey.ac.nz (S.M.); c.a.wham@massey.ac.nz (C.W.); 2 Department of Sport Sciences and Physical Education, Sabaragamuwa University, Belihuloya 70140, Sri Lanka; 3 Office of the Vice-Chancellor, AUT University, Auckland 1010, New Zealand; elaine.rush@aut.ac.nz; 4 Riddet Institute, Massey University, Palmerston North 4442, New Zealand

**Keywords:** dietary intake assessment, 24 h multiple pass recall, weighed food diary, fruit and vegetable, Veggie Meter^®^, carotenoid

## Abstract

Adequate intake of fruits and vegetables (F + V) supports healthy growth and development in children, yet many New Zealand children do not meet national dietary recommendations, and methods to evaluate intake require good reliability. **Objectives:** To establish the validity and reliability of a modified 24 h multiple pass recall (MPR) for evaluating F + V and carotenoid intakes in children aged 9–13 years. The reliability of the Veggie Meter^®^ (VM^®^), a non-invasive reflection spectrometer to estimate skin carotenoid scores and derive blood carotenoid concentrations, was also examined. **Methods:** Thirty-two children (20 boys, 12 girls) completed three 24 h MPRs and parent-assisted weighed food diaries (WFDs) on randomised weekdays and weekends. Skin carotenoid scores were assessed using the VM^®^. The validity of the MPR was evaluated against WFDs using log-transformed Pearson correlations and mean x-axis bias. The reliability was assessed by the coefficient of variation (CV) and Pearson correlations. **Results:** Participants did not meet recommended F + V intakes (5–5.5 servings/day): MPR (mean fruit 1.3 servings/day; vegetables 2.0), WFD (fruit 1.3; vegetables 1.9). The MPR was a valid tool to estimate fruit and vegetable daily servings (combined-day Pearson coefficients > 0.71) with only trivial–small standardized mean bias-offset vs. WFD; however, the reliability was poor for the MPR-estimated carotenoid intake (CV 126%) and F + V intake. In contrast, the VM^®^ was reliable (Pearson correlation 0.97–0.99) with low measurement error (CV 4.0–5.2%). **Conclusions:** The modified 24 h MPR was valid but unreliable for estimating F + V and carotenoid intake. The VM^®^ demonstrated high reliability as a biomarker of skin carotenoid status in children.

## 1. Introduction

The last National Children’s Nutrition Survey in New Zealand was conducted over 20 years ago [1]. Since then, substantial demographic and socioeconomic changes have occurred, including increased ethnic diversity—with a growing proportion of the population identifying as Māori, Pacific, and Asian [2]—along with shifts in food availability, food preferences, and the affordability of healthy food options [3]. These shifts may substantially affect the current dietary habits and nutritional health of children [3]. Therefore, an updated understanding of children’s food intake, particularly fruit and vegetable (F + V) consumption, is urgently needed.

Fruits and vegetables are essential components of a healthful diet and have been consistently associated with improved growth and reduced risk of obesity, heart disease, diabetes, and certain cancers [4,5]. Despite this, most New Zealand children aged 9–13 years do not meet national F + V intake recommendations of 5–5.5 servings of vegetables and at least 2 servings of fruit each day [6]. The most recent New Zealand Health Survey (2022/23) found that only 5.4% of children met the vegetable intake recommendation, while 70.9% met the fruit intake recommendation [7], pointing to the need for further evaluation with valid and reliable tools.

The 2002 National Survey used a 24 h multiple pass recall (MPR) to assess dietary intake [1]. While the MPR is widely used in nutrition surveillance, its accuracy depends on the age and recall ability of the participant. Children may lack knowledge of brand names, preparation methods, and portion sizes. To address these limitations, we have developed a modified 24 h MPR that includes parental input to improve completeness and accuracy and visual aids such as standardised household measures and a ‘Photographic Atlas of Food Portion Sizes’ [8]. Herein, we aim to determine if the current modified 24 h MPR improves the reliability and validity of F + V intake estimates in children.

In addition to recall-based dietary assessments, non-invasive biomarker tools such as the Veggie Meter^®^ (VM^®^) may offer objective measures of F + V intake. The VM^®^ uses reflection spectroscopy to quantify skin carotenoid scores (SCSs), which correlate directly with the concentration of carotenoid in the blood and strongly with habitual F + V intake [9,10,11,12,13]. Carotenoids are a biomarker for F + V consumption due to the high concentrations in these foods and their inability to be synthesised by the body. Although the VM^®^ has shown promise in adult populations, its intra- and inter-day reliability and feasibility for use in school-aged children has not yet been established. This question provides the second primary aim of the current study and, together with the first aim, will provide information for the design of larger-scale dietary assessments in children and yield updated estimates of F + V quantitative intake and variability, aiding in sample size calculations for future research.

## 2. Materials and Methods

### 2.1. Study Design

**Figure 1:**
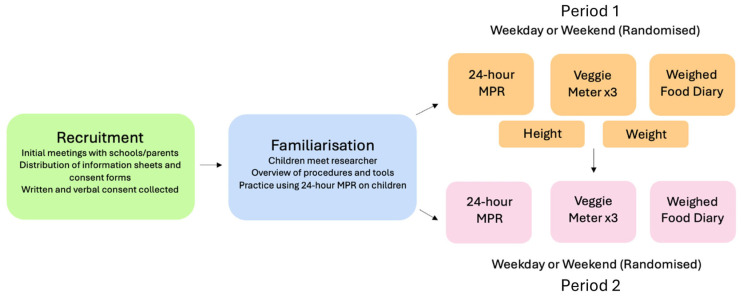
Research design, sampling days, and assessment procedures in both arms (Periods 1 and 2) of the crossover. 24 h MPR, modified 24 h multipass recall.

This study was a randomised observational study comprising a weekday and weekend (a) modified 24 h MPR completed by the child, (b) a WFD for the same 24 h period completed by the child’s parent or caregiver, and (c) VM^®^ assessments (Figure 1). The WFD provided the only practical way to qualitatively and quantitatively assay free-living food intake of the children and therefore was used as the criterion reference method to establish an estimate of the validity and reliability of the modified 24 h MPR.

Following information and consent, the child–parent/caregiver pair were familiarised with the procedures and tools (Figure 1). Thereafter, each pair was randomly assigned to the assessment of either a weekday school-day first, followed by a weekend day, or vice versa over a one-to-three-week period. Participating children and schools received NZD 75 and NZD 200 vouchers, respectively, on completion, as a koha/gift of gratitude and in part to reimburse for school staff time contributions and the use of their facilities for interviews.

### 2.2. Ethics

Children and parents/caregivers were informed of the study through written and verbal discourse. Children gave verbal and their parents/caregivers written consent. Researchers underwent police vetting to ensure child safety. All procedures were reviewed and approved by the Health Research Council accredited Massey University Human Ethics Committee, Ohu Matatika 1, Application OMI 23/46.

### 2.3. Participants, Sample Size, and Recruitment

Participants aged 9–13 years living in Auckland, New Zealand, were primarily recruited through schools and affiliated networks, as well as flyers in community locations and posts on Facebook community groups. This age range was selected as evidence suggests that from the age of nine, children can reliably complete a 24 h MPR with minimal adult support [14]. Inclusion criteria included residence and school attendance in Auckland and a parent/caregiver willing and able to complete a WFD following researcher training. Recruitment initially targeted schools with high equity index values—indicative of greater socioeconomic barriers to learning—but all except two declined participation; one yielded five volunteers, and the other yielded none. Additional schools were subsequently contacted by email and phone.

A minimum sample size of 31 participants for this study was determined based on the intraclass correlation coefficient (ICC) for weighed and modified 24 h MPRs of 0.98 for energy, 0.97 for protein, and 0.94 for iron [15]. Assuming the minimum acceptable ICC of 0.9 [16,17], 25 children would be required [18]. In an analysis of the expected validity of the 24 h MPR vs. WFD, the 36 kcal (2.8%) difference in energy between the measure and criterion, with SD 409 kJ, SE 14 kJ, and within-subject SD (typical error) 47 kJ, required a sample size of 26 at 90% power and 5% type-1 error. Out of the two sample sizes, the larger one was selected (n = 26) to ensure statistical power and precision. Accommodating an estimated 20% dropout, a minimum sample size of 31 was selected.

### 2.4. Familiarisation

Familiarisation was led by trained registered or student dietitians and comprised introducing and familiarising the child with the modified 24 h MPR and training the parent in completing the WFD that included the extent of detail required for qualitative and quantitative accuracy. Parents were also asked to fill out a VM^®^ questionnaire regarding their child’s intake of F + V, supplements, and other foods high in vitamin A (Supplementary File S1). Following familiarisation, child–parent/caregiver pairs were offered the opportunity to ask questions and then were provided with the measuring equipment, as described below.

### 2.5. Modified 24 h MPR

The modified 24 h MPR was adapted from the National Children’s Nutrition Survey [1]. The modifications included the following: (a) a familiarisation process prior to the interviews, (b) parental support in the third phase of the recall, (c) the use of the Photographic Atlas of Food Portion Sizes [8] adapted for use in Life and Living in Advanced Age: A Cohort Study in New Zealand (LiLACS NZ), and (d) standardised measuring equipment to assist with portion estimations and conducting the recall without computer assistance.

The modified 24 h MPRs were undertaken with the children at either the home, school classroom, or in a meeting room at the University. The first 24 h MPR session also included measuring the participating child’s weight and height using a portable weight scale and stadiometer. Each session took approximately 20–30 min.

One weekday and one weekend modified 24 h MPR were completed on the day after each respective WFD. In the first pass (Supplementary File S2), the researcher interviewed the child and recorded an uninterrupted quick list of their food and drink intake. In the second pass (Supplementary File S3), a detailed list of the child’s food and drink intake was recorded; this included specifics such as time, place, brand, and amount, which was aided using the Photographic Atlas and measuring equipment. The third pass consisted of reviewing the food and drink intake with the child and parent/caregiver to fill in any gaps by asking open-ended questions and recording any recipes or brands that were provided.

### 2.6. Weighed Food Diary

Following recruitment and familiarisation, parents or caregivers were provided with written instructions, scales, measuring spoons and cups, and a printed copy of the data entry booklet (Supplementary File S4). In addition, digital photographs of all the food and drinks (including uneaten food) were taken and later forwarded to the independent researchers. Information on food and drinks purchased at school was obtained by asking the children to explain what they ate or to retain packaging for their parents to record. The WFD results were discussed and collected following the completion of both modified 24 h MPRs to minimise carryover bias.

### 2.7. Veggie Meter^®^

The VM^®^ measures SCSs in the fat pad under the skin [19]. Before use, the VM^®^ was calibrated using dark and light reference materials; repeat calibrations occurred every hour. Participants were required to wash and dry their hands thoroughly. Their right index finger pad was then placed facing down on the bulb of the VM^®^. The average of three VM^®^ SCS samples were collected providing scans between 0 and 800 with an allowance of less than 10% variability between each score and used to assess the accuracy of the modified 24 h MPR and WFDs for assessing dietary F + V intake. The VM^®^ SCSs were converted to plasma carotenoid concentration VM^®^ dCC (μmol/L) through the following equation: 0.0056 VM^®^ SCS [20].

### 2.8. Data Processing and Analysis

Ingested food and drink items within the diaries were processed within FoodWorks V.2.0 [21] by two different researchers independent of those conducting the MPRs. Foods unavailable within the software were manually entered using nutrition panels. The New Zealand food and nutrition guidelines for healthy children and young people aged 2–18 years were used to determine F + V serving size [22]. Examples of one serving size of F+V included 130 g of apple, banana, or orange; 135 g of canned fruit; 50–80 g of cooked vegetables; 135 g of potato or kumara; and 60 g salad [22]. The total carotenoid intake was calculated from the sum of lycopene, lutein, zeaxanthin, β-carotene, α-carotene, and β-cryptoxanthin generated within the software [21].

### 2.9. Statistical Analysis

The validity and reliability for outcomes was estimated in a spreadsheet [17]. The validity of the modified 24 h MPR to estimate fruit, vegetable, and carotenoid intake was compared against the criterion values obtained from the WFD. All data except F + V servings due to zero values were 100*Ln-transformed to manage heteroscedasticity apparent on visual inspection and examination of skewness and kurtosis of the residuals, where >±2 and >±7 were considered nonnormal [23], and to promote uniformity of error and validity estimated by Pearson correlation and standardised bias at *x* (axis) derived from regression analysis [24]. Standardised bias at x evaluated the magnitude of the difference between the modified 24 h MPR and WFDs. It was calculated by dividing the mean bias of the modified 24 h MPR by the standard deviation of the WFD; this measure indicated overestimation when the bias was positive, while a negative bias suggested underestimation, with the magnitude interpreted using a modified Cohen scale [25]. The Pearson correlation measures the similarity between two variables [24]. The magnitude thresholds for Pearson correlations were ≥0.1 = small, ≥0.3 = moderate, ≥0.5 = large, ≥0.7 = very large, and ≥0.9 = extremely large [25].

The reliability of the modified 24 h MPR fruit, vegetable, and total F + V serving intake was expressed as the inter-day (weekday vs. weekend days) difference between the within-day 24 h MPR serving minus WFD serving estimates (i.e., difference of the difference); this approach was taken over log-transformation because of the zero values for some samples between the two forms of diaries and the fact that zero cannot be log-transformed. The inter-day reliability of the modified 24 h MPR carotenoid intake (μg/day) was estimated from the 100*Ln of the 24 h MPR/WFD ratio. The inter-day values for the VM^®^ dCC were also log-transformed. The derived reliability statistics were the percentage change in the mean, mean typical error as a CV (%), and Pearson correlation. The CV is presented as the mean error score of the estimated intake and measures the within-subject variability present within repeated measurements [17]. A smaller typical error indicated more consistency, while a larger typical error indicated greater inconsistencies [16]. The standardised typical error of estimate was calculated by taking the single estimate divided by the sample variation (standard deviation); due to brevity, these data are not shown in the main text (see worksheets in Supplementary File S14) but were used to qualify the effect magnitude to aid in inferential description of the statistic.

The modified Cohen scale was used to interpret the magnitude as follows: for standardised typical error <0.1 = trivial, 0.1–0.3 = small, 0.3–0.6 = moderate, 0.6–1.0 = large, 1.0–2.0 = very large, and >2.0 = extremely large. The thresholds for the mean x-axis bias and the standardised raw change in mean and standardised raw typical error for reliability were 0.2 = small, 0.6 = moderate, 1.2 = large, 2.0 = very large, and 4.0 = extremely large [26].

## 3. Results

### 3.1. Participants and Data

We obtained more interest than expected, resulting in thirty-seven participants being recruited and enrolled in this study, which, after dropout, left 32 completing the study. Three participants dropped out due to personal reasons, and the data from two participants were removed due to incorrect procedures (Supplementary File S5). All data and analysis have been provided in Supplementary Files S12–S15.

### 3.2. Carotenoids

The recommended average daily carotenoid intake is 5340 μg/day for boys and 5040 μg/day for girls [27]. With 24 h MPR intakes of (mean ± SD) 3045 ± 3535 μg/day for boys and 2493 ± 3698 μg/day for girls and WFD intakes of 2816 ± 2983 for boys and 1974 ± 2263 for girls on average, neither boys nor girls met the requirement, but there was wide interindividual variation. Three girls met the carotenoid intake for the weekday and weekend 24 h MPR, while seven boys met it. For the weekday and weekend WFD intakes, two girls and six boys met the recommended intake.

### 3.3. Vegetable Intake

**Figure 2:**
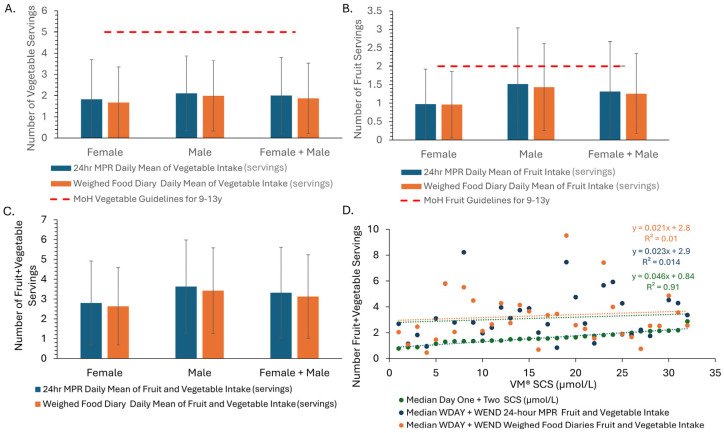
(**A**) Mean daily vegetable intake as estimated from the modified 24 h multipass recall (MPR) and WFD. (**B**) Mean daily of fruit intake as estimated from the modified 24 h MPR and WFD. For panels A and B, the red dashed line is the NZ Ministry of Health Guidelines for Vegetable Servings for 9- to 13-year-olds [28]. (**C**) Daily mean fruit and vegetable intake as estimated from the modified 24 h MPR fruit and WFD. (**D**) VM^®^ dCC vs. median child-sample value for fruit and vegetable intake estimated from the modified 24 h MPR and WFDs. Histograms are raw means, and error bars represent standard deviations. MOH, Ministry of Health; WFD, weighed food diary; VM^®^, Veggiemeter spectrophotometer; dCC, derived carotenoid concentration.

Both boys and girls had an average intake of less than 5–5.5 vegetable servings/day, although boys had a higher vegetable intake than girls (Figure 2A). Vegetable intake was overestimated in the modified 24 h MPR when compared to the WFD (Figure 2A). Children’s vegetable intake for the weekday modified 24 h MPR and WFDs was higher than their vegetable intake for the weekend (Supplementary Files S6–S11).

### 3.4. Fruit Intake

On average, boys and girls had an intake of less than two fruit servings a day for the weekday and weekend modified 24 h MPR and WFDs; however, boys had an overall higher fruit intake than girls (Figure 2B). Fruit intake was overestimated by both girls and boys when comparing the modified 24 h MPR to the WFD (Figure 2B).

### 3.5. Total Fruit and Vegetable Intake

Total F + V intake for the weekday modified 24 h MPR and WFD had an overall higher estimated intake than the weekend (Supplementary Files S6–S11). Boys had an overall higher F + V intake than girls, but neither group met the recommended intake (Figure 2C). Total F + V intake was overestimated in both girls and boys for the modified 24 h MPR (Figure 2C).

### 3.6. Skin Carotenoid Score vs. Median F + V Intake from the Modified 24 h Multiple Pass Recall and Weighed Food Diaries

Figure 2D shows that there was no relationship between VM^®^ dCC, the modified 24 h MPR, and the WFD F + V intake. The relationship between the modified 24 h MPR and WFD shows some similarity (Figure 2D).

### 3.7. Validity of the Modified 24 h Multiple Pass Recall

**Figure 3:**
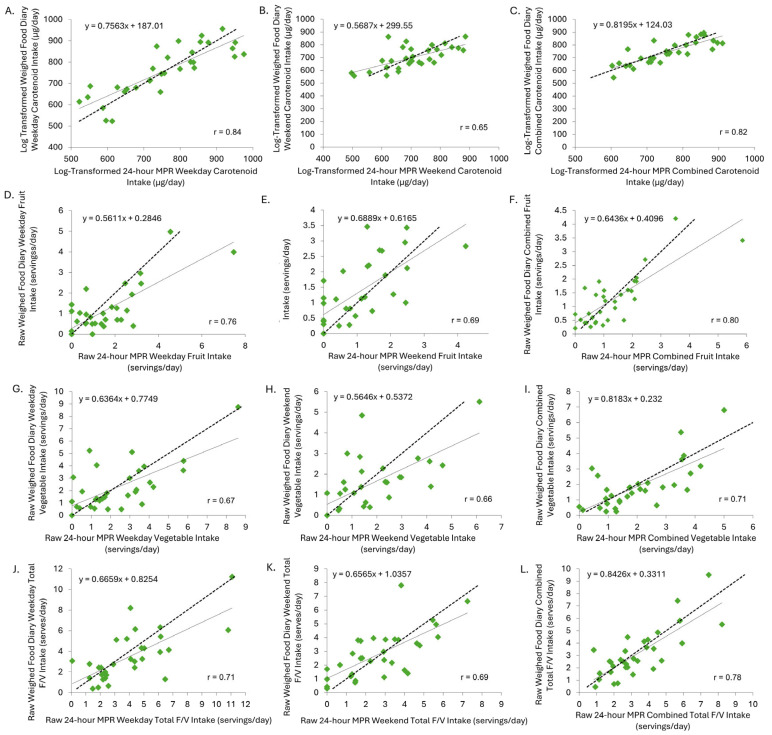
(**A**) Correlation between log-transformed 24h MPR and weighed food weekday carotenoid intake (μg/day). (**B**) Correlation between log-transformed 24 h MPR and weighed food weekend carotenoid intake (μg/day). (**C**) Correlation between log-transformed 24 h MPR and weighed food combined carotenoid intake (μg/day). (**D**) Correlation between raw 24 h MPR and weighed food weekday fruit intake (servings/day). (**E**) Correlation between raw 24 h MPR and weighed food weekend fruit intake (servings/day). (**F**) Correlation between raw 24 h MPR and weighed food combined fruit intake (servings/day). (**G**) Correlation between raw 24 h MPR and weighed food weekday vegetable intake (servings/day). (**H**) Correlation between raw 24 h MPR and weighed food weekend vegetable intake (servings/day). (**I**) Correlation between raw 24 h MPR and weighed food combined vegetable intake (servings/day). (**J**) Correlation between raw 24 h MPR and weighed food weekday total F + V intake (servings/day). (**K**) Correlation between raw 24 h MPR and weighed food weekend total F + V intake (servings/day). (**L**) Correlation between raw 24 h MPR and weighed food combined total F + V intake (servings/day). Dashed line is line of identity. Solid line is the model regression line. 24 h MPR, 24 h multiple pass recall.

There were large to very large mean positive correlations between the 24 h MPR and WFD for carotenoid intake, total fruit servings, vegetable servings, and total F + V servings (Table 1; Figure 3A–L). The 24 h MPR consistently showed trivial–small positive bias for fruit and vegetable intake and carotenoids across all days (Table 1).

### 3.8. Reliability of the Modified 24 h MPR and Veggie Meter^®^

**Figure 4:**
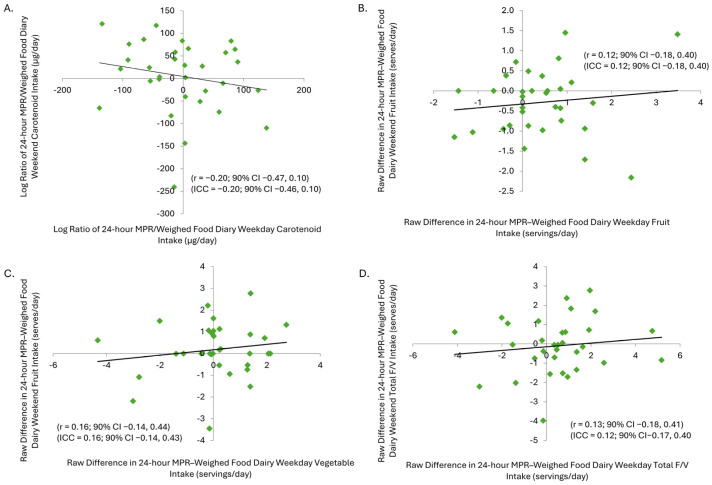
(**A**) Correlation between log-transformed ratio of 24 h MPR/WFD on weekday and weekend for carotenoid intake as a measure of reliability. (**B**) Correlation between raw difference in 24 h MPR–WFD servings on weekday and weekend for fruit intake as a measure of reliability. (**C**) Correlation between raw difference in 24 h MPR–WFD diary on weekday and weekend for vegetable intake. (**D**) Correlation between raw difference in 24 h MPR–WFD on weekday and weekend for total F + V intake. The black line is the regression line. 24 h MPR, 24 h multiple pass recall; WFD, weighed food diary.

The modified 24 h MPR showed large typical error for carotenoid intake and moderate to large error for fruit, vegetable, and total F + V intake (Table 2), with varied reliability outcomes. There was a weak negative correlation for carotenoid intake and weak positive correlations for fruit, vegetable, and total F + V intake (Table 3). The modified MPR showed poor positive Pearson correlations for carotenoid intake and poor Pearson correlations for fruit, vegetable, and total F + V intake overall (Table 2 and Table 3; Figure 4A–D). Vegetable intake showed a small change in mean, whereas total F + V intake exhibited a very large standardised raw change in mean (Table 2). For the VM^®^, the percentage change in mean for weekday, weekend, combined, and between-day dCC was small, indicating excellent reproducibility. Typical error was small for the VM^®^ across all time points (Table 3). The Pearson correlations reinforced these findings (Table 3; Figure 4A–D).

## 4. Discussion

The current study evaluated the validity and reliability of a modified 24 h MPR for assessing fruit, vegetable, and carotenoid intake in New Zealand children aged 9–13 years. Additionally, the inter-day reliability of the VM^®^, a non-invasive spectrophotometric tool for assessing SCSs and dCC, was examined. The key findings were that the modified 24 h MPR was a valid but not very reliable tool for assessing F + V and carotenoid intake, while the VM^®^, in contrast, demonstrated excellent reliability across all tested parameters.

### 4.1. Validity of the Modified 24 h MPR

Large to very-large positive correlations and trivial–small positive bias against the WFD values for carotenoid, fruit, vegetable, and total F + V intake (Table 1) confirmed the validity of the modified 24 h MPR as a dietary assessment tool for our sample of children aged 9–13 years. These findings are consistent with previous literature showing that multiple 24 h recalls improve validity compared to single-day recalls in children [29].

### 4.2. Correlation Between F + V Intake and Skin Carotenoid Scores

In contrast to prior studies that demonstrated positive associations between SCSs and F + V intake using food frequency questionnaires (FFQs) [12,13], our study found no clear correlation between SCSs measured by the VM^®^ and F + V or carotenoid intake estimated by the 24 h MPR. Sampling variability is one explanation; another is the disparity in measurement time windows. The VM^®^ measures the SCS as a chronic biomarker reflecting the stable skin carotenoid reservoir [30]. Carotenoids are slowly incorporated into the skin (detectable increase after 2 to 6 weeks of sustained intake [31]) and clear slowly, with a half-life of several months [32]. Furthermore, while FoodWorks is a widely used food composition database standard [integrating Australian (AUSNUT) and United States (USDA) food composition data], the inherent measurement variability of nutrient values in these databases vs. ingested, coupled with the 24 h MPR method’s acute nature, may have further compromised the accuracy [33]. Additionally, some carotenoids such as lycopene are found in few foods, with most coming from tomatoes and tomato products [34], making the top food sources of lycopene pizza, spaghetti, lasagna, and ketchup. Thus, lycopene is not considered a biomarker of total fruit and vegetable intake but rather tomato product intake [31], with implications for inference to dietary prescription to raise carotenoid intake.

Meanwhile, other authors reported small but significant correlations between SCSs and total F + V intake in larger cohorts of preschool-aged children [12,13]. These findings highlight the need for replication in larger New Zealand samples and suggest that pairing the VM^®^ with an FFQ or more days of sampling, rather than 1 or 2 MPRs, may enhance convergent validity. Despite the lack of observed correlation in this study, our observed high reliability and other existing research supports the use of the VMR as a valid biomarker of chronic carotenoid intake [4,9,10,11].

### 4.3. Reliability of the Modified MPR

The modified 24 h MPR showed poor inter-day reliability for estimated F + V and carotenoid intakes (Table 2). Large changes in mean and moderate to very-large high typical error reflected inconsistency in children’s reporting, likely due to their limited role in meal selection and recall. The Pearson and intraclass correlations were weak for all intake outcomes, suggesting low day-to-day reproducibility.

These findings are supported by St. George, et al. [35], who reported that even three repeated 24 h dietary recalls yielded low reliability in children. They estimated that up to 21–32 recalls would be needed to achieve 80% accuracy for fruit intake—clearly not practical in field research. In this study, the unreliability could also reflect natural intake variation between weekdays and weekends. During the week, many children have a set lunch box routine that usually consists of a main meal, fruit, and snacks, However, on weekends at home, meals may vary more, and fruit is offered less consistently, making intake less structured and controlled. Additionally, there will be some additional error associated with the WFD assessment, but this was not quantified in the current methodology.

Despite poor reliability, the magnitude of measurement error provides useful information for sample size estimation in future intervention studies. Based on within-subject standard deviations (TEE = 126%) and between-subject SDs for carotenoid intake (μg), a parallel-groups trial designed to detect moderate effects (0.6 × SD) would require 41 participants per group using standard alpha = 5% and beta = 80% error rates, which is a feasible target depending on study resources.

### 4.4. Reliability of the Veggie Meter^®^

In contrast to the MPR, the VM^®^ demonstrated excellent inter-day reliability for SCS and dCC values. Log-transformed Pearson correlations were high (0.97–0.99), and typical errors as CVs were low across all conditions (Table 3), confirming the VM^®^ as a consistent measure of tissue carotenoids. These findings align with previous studies in children and adolescents.

Interestingly, in this study, girls had higher SCSs than boys, despite reporting lower F + V and carotenoid intake. This is inconsistent with prior research showing that boys generally consume more F + V and have higher SCSs [36]. Our findings were more consistent with Takeuchi, et al. [37], who reported higher SCSs in girls. This discrepancy may reflect the need for a longer-duration of MPR sampling as noted above, sampling variability, gender differences in body composition, or behavioural factors not captured in dietary assessment and warrants further exploration.

### 4.5. Overestimation and Reporting Bias

Overestimation of F + V intake was observed in both boys and girls, reflecting a desire to appear healthier, a known limitation of self-reported dietary assessment [38,39]. Despite training and visual aids, children in this age group may lack the attention to detail or interest in recording intake accurately, especially when foods are prepared for them; additionally, the data may also point to another contribution in that most parents either do not know or are unable to provide the appropriate number of F + V portions. This suggests that the modified 24 h MPR, while feasible and valid, may still require adjustment for overreporting or need to be supplemented with objective tools such as the VM^®^ or wearable cameras.

### 4.6. Strengths and Limitations

A strength of this study was the structured use of portion estimation tools, including the LiLACS NZ Photographic Atlas of Food Portion Sizes and standardised household measures. The inclusion of parental input also helped improve data completeness.

The limitations include the small although a priori adequate sample size, which reduced the power for detecting correlations, and the reliance on WFDs as a reference method—subject to its own biases such as parental over-observation, which meant that there was a high likelihood of alterations being made to the children’s diet to make their intake more healthy or appealing. Various items in the LiLACS NZ Photographic Atlas of Food Portion Sizes may also be outdated and not fully reflective of current food environments or multicultural diets in New Zealand. Additionally, natural day-to-day variability in diet was not controlled, which may have inflated within-subject variation. Another limitation to be noted is that carotenoid intake is not only influenced by fruits and vegetables; therefore, a high VM^®^ SCS does not necessarily mean a high F + V intake.

## 5. Conclusions and Future Directions

This study found that the modified 24 h MPR was a valid but poorly reliable tool for estimating F + V and carotenoid intakes in a sample of New Zealand children aged 9–13 years. The tool showed excellent precision for serving size estimation, but the low reliability suggests its use should be approached cautiously with an adequate sample size. In contrast, the VM^®^ demonstrated high reliability, suggesting its utility as a non-invasive biomarker of low carotenoid status and, therefore, carotenoid-containing fruits and vegetables, within this age group.

Future research should assess the modified 24 h MPR’s reliability in larger and more diverse cohorts. Comparisons with FFQs and the integration of emerging technologies—such as wearable cameras—may enhance intake tracking but raise ethical and logistical considerations. Food nutrient composition databases require updating as the fundamental biomarker of intake. Differences in F + V intakes between genders also merit further investigation. Finally, large-scale studies are needed to validate the VM^®^ as a tool to track dietary intervention effects and to further refine child-specific protocols for its application.

## Figures and Tables

**Table 1:** Validity measures for estimates of fruit, vegetable, and carotenoid intake derived from the 24 h MPR.

Attribute	Sample	Pearson Coefficient	Mean Standardised Bias at x (Predictor) Value
Total Fruit (servings)	WDAY:	0.76 (0.61, 0.86)	0.35 (0.09, 0.61)
	WEND:	0.69 (0.49, 0.82)	−0.28 (−0.51, −0.04)
	COMB:	0.80 (0.66, 0.89)	0.06 (−0.16, 0.29)
Total Vegetable (servings)	WDAY:	0.67 (0.46, 0.81)	0.04 (−0.21, 0.29)
	WEND:	0.66 (0.45, 0.80)	0.14 (−0.14, 0.41)
	COMB:	0.71 (0.53, 0.83)	0.09 (−0.13, 0.30)
Total Fruit + Vegetable (servings)	WDAY:	0.71 (0.52, 0.83)	0.20 (−0.03, 0.44)
	WEND:	0.69 (0.50, 0.82)	−0.06 (−0.30, 0.18)
	COMB:	0.78 (0.63, 0.88)	0.10 (−0.09, 0.29)
Carotenoids (μg)	WDAY:	0.84 (0.72, 0.91)	−0.02 (−0.29, 0.24)
	WEND:	0.65 (0.44, 0.79)	0.06 (−0.35, 0.47)
	COMB:	0.82 (0.69, 0.90)	0.15 (−0.09, 0.39)

Fruit, vegetable, and fruit + vegetable statistics were derived from raw data, and carotenoid statistics were derived from log-transformed raw data. Data are mean estimates (90% CLs). WDAY, weekday; WEND, weekend; COMB, combined (average of weekday and weekend); MPR; multiple pass recall. Thresholds for mean standardised raw bias at x value for validity were as follows: <0.2 = trivial, 0.2–0.6 = small, 0.6–1.2 = moderate, 1.2–2.0 = large, 2.0–4.0 = very large, and >4.0 = extremely large.

**Table 2:** Reliability measures for the modified 24 h MPR.

Attribute (Servings)	Standardised Raw Change in Mean	Standardised Mean Raw Typical Error (90% CL)	Mean Raw Pearson Correlation
Modified 24 h MPR vs. Weighed Fruit Intake	−2.2 (−3.4, −1.0)	0.94 (0.78, 1.2)	0.12 (−0.18, 0.40)
Modified 24 h MPR vs. Weighed Vegetable Intake	−0.2 (−0.8, 1.2)	0.92 (0.76, 1.2)	0.16 (−0.14, 0.44)
Modified 24 h MPR vs. Weighed F + V Intake	−1.0 (−2.2, 0.1)	0.94 (0.78, 1.2)	0.13 (−0.18, 0.41)

Data are mean estimates (90% CLs). WDAY, weekday; WEND, weekend; COMB, combined (average of weekday and weekend); MPR, multiple pass recall. Thresholds for raw change in mean and raw typical error for reliability were as follows: 0.2 = small, 0.6 = moderate, 1.2 = large, 2.0 = very large, and 4.0 = extremely large.

**Table 3:** Reliability Measures for the Veggie Meter^®^ and the carotenoid intake estimated from the modified 24 h MPR.

	Statistic		
Attribute	Change in Mean (%)	Typical Error as a CV (%)	Pearson Correlation
Modified 24 h MPR Carotenoid Intake (μg)	8 (−23, 53)	126 (97, 181)	−0.20 (−0.47, 0.10)
Weekday VM^®^ SCS	2-1: 1.0 (−0.6, 2.7)	2-1: 4.0 (3.3, 5.1)	2-1: 0.97 (0.97, 0.99)
	3-2: 1.0 (−1.5, 3.6)	3-2: 6.2 (5.1, 8.0)	3-2: 0.96 (0.93, 0.98)
Weekend VM^®^ dCC (μmol/L)	2-1: 1.0 (−0.7, 2.7)	2-1: 4.1 (3.4, 5.2)	2-1: 0.98 (0.97, 0.99)
	3-2: 1.2 (−0.4, 2.8)	3-2: 3.8 (3.2, 4.9)	3-2: 0.99 (0.97, 0.99)
Combined VM^®^ dCC (within day) (μmol/L)	2-1: 1.0 (−0.6, 2.7)	2-1: 4.0 (3.3, 5.1)	2-1: 0.98 (0.97, 0.99)
	3-2: 1.0 (−1.5, 3.6)	3-2: 6.2 (5.1, 8.0)	3-2: 0.96 (0.93, 0.98)
	4-3: −0.5 (−3.2, 2.3)	4-3: 6.7 (5.5, 8.5)	4-3: 0.96 (0.92, 0.98)
	5-4: 1.0 (−0.7, 2.7)	5-4: 4.1 (3.4, 5.2)	5-4: 0.98 (0.97, 0.99)
	6-5: 1.2 (−0.4, 2.8)	6-5: 3.8 (3.2, 4.9)	6-5: 0.99 (0.97, 0.99)
Between Weekday and Weekend VM^®^ dCC (μmol/L)	2-1: 1.5 (−0.2, 3.3)	2-1: 4.2 (3.5, 5.3)	2-1: 0.98 (0.97, 0.99)
	3-2: −0.5 (−2.2, 1.2)	3-2: 4.2 (3.5, 5.4)	3-2: 0.98 (0.97, 0.99)
	4-3: 1.5 (0.0, 3.0)	4-3: 3.5 (2.9, 4.5)	4-3: 0.99 (0.98, 0.99)
	5-4: −0.5 (−2.9, 2.1)	5-4: 6.2 (5.1, 7.9)	5-4: 0.96 (0.93, 0.98)
	6-5: 1.7 (−0.7, 4.1)	6-5: 5.8 (4.8, 7.4)	6-5: 0.97 (0.94, 0.98)

All statistics were derived from log-transformed raw data. Number contrasts (e.g., 2-1) denote the number of the repeated test. WDAY, weekday; WEND, weekend; COMB, combined (average of weekday and weekend); VM^®^, Veggie Meter^®^; dCC, derived carotenoid concentration. Thresholds for log-transformed raw change in mean for reliability were as follows: 0.2 = small, 0.6 = moderate, 1.2 = large, 2.0 = very large, and 4.0 = extremely large. Values are 90% CI. #-# = VM^®^ dCC test compared to each other.

## Data Availability

The analysis outcomes are in Supplementary Data Files S1–S11. The data spreadsheets are in Supplementary File S12; the validity and reliability analysis spreadsheets are in S13 and S14, respectively; and the VM^®^ data spreadsheet is in Supplementary File S15.

## Supplementary Materials

The following supporting information can be downloaded at: https://www.mdpi.com/article/10.3390/nu17203293/s1: Supplementary Files S1–S15.

## Abbreviations

The following abbreviations are used in this manuscript:

BMI	Body Mass Index
CV	Coefficient of Variation
FFQ	Food Frequency Questionnaire
F + V	Fruits and Vegetables
ICC	Intraclass Correlation Coefficient
MUHEC	Massey University Human Ethics Committee
MELAA	Middle Eastern, Latin American, African
MPR	Multiple Pass Recall
WFD	Weighed Food Diary
NSLP	National School Lunch Program
NCDs	Noncommunicable diseases
RS	Reflection Spectroscopy
RRS	Resonance Rama Spectroscopy
SPAN	School Physical Activity and Nutrition
STEAM	Science, Technology, Engineering, Arts, and Math
SCS	Skin Carotenoid Score
dCC	Derived Carotenoid Concentration
TEE	Typical Error of Estimate
VM^®^	Veggie Meter^®^
