# Supplementary material for: Reliability of a Modified 24 h Dietary Recall and Veggie Meter to Assess Fruit and Vegetable Intake in New Zealand Children
Source: Nutrients. 2025 Oct 20;17(20):3293. doi: 10.3390/nu17203293 (PMC12566992; doi:10.3390/nu17203293)
Supplement: Supplementary file 1 [file nutrients-17-03293-s001.zip › nutrients-3890880-Files S1-S11.pdf]

Supplementary Materials

Reliability of a Modified 24 h Dietary Recall and Veggie Meter to Assess Fruit and Vegetable Intake in New Zealand Children

Varshika V. Patel [1], Thalagalage Shalika Harshani Perera [1,2], Elaine Rush [3,4], Sarah McArley [1], Carol Wham [1] and David S. Rowlands [1,4,*]

[1] School of Sport, Exercise and Nutrition, Massey University, Auckland 0632, New Zealand; varshika.vpatel@gmail.com (V.V.P.); shalika.harshani@gmail.com (T.S.H.P.); s.m.mcarley@massey.ac.nz (S.M.); c.a.wham@massey.ac.nz (C.W.)

[2] Department of Sport Sciences and Physical Education, Sabaragamuwa University, Belihuloya 70140, Sri Lanka

[3] Office of the Vice-Chancellor, AUT University, Auckland 1010, New Zealand; elaine.rush@aut.ac.nz

[4] Riddet Institute, Massey University, Palmerston North 4442, New Zealand

* Correspondence: d.s.rowlands@massey.ac.nz; Tel. +64-272099383

Contents

Figure S1. Questionnaire for Veggie Meter Foods...................................... 3

Figure S2. The Modified 24-hour MPR First Pass... 3

Figure S3. The Modified 24-hour MPR Second Pass.................................... 4

Figure S4. Weighed Food Diary.. 4

Figure S5 Participant characteristics... 4

Figure S6. Weekday Modified 24-hour MPR Food Intake............................. 5

Figure S7. Weekend Modified 24-hour MPR Food Intake............................. 6

Figure S8. Average of Weekday and Weekend Modified 24-hour MPR Food Intake 6

Figure S9. Weekday Weighed Food Diary Intake....................................... 7

Figure S10. Weekend Weighed Food Diary Intake...................................... 8

Figure S11. Average of Weekday and Weekend Weighed Food Diary Intake.... 8

Questionnaire for Veggie Meter Foods

(The researcher will fill this out when the Veggie meter reading is taken.)

Date: …………………………

01. Age: ……………..

02. Height: …………….cm

03. Weight: ………………kg

04. Gender: ………………..

05. Ethnicity: ………………

Choose the <u>best</u> answer by placing a circle around it according to the child's answer.

06	Are there any **foods that you <u>do not</u>** eat?	Yes		No		Don't Know
	If yes, what are they?					
07	Do you eat **liver** (including liver pate)?	Yes		No		Don't Know
	If yes, how often	every day	most days	every week	every month	once a year
08	Do you eat **eggs**?	Yes		No		Don't Know
	If yes how often	every day	most days	every week	every month	once a year
09	Do you take any **vitamin supplements**?	Yes			No	
	If yes, name the supplement					
	If yes how often					

Vegetables and fruits

A serving is a handful. It can be fresh, frozen or canned. Do not count fries or hot chips as a vegetable.

10	How many servings of **vegetables** do you eat **in one day?**									
	0	1	2	3	4	5	6	7	7+	Don't Know
11	How many servings of **fruit** do you eat **in one day?**									
	0	1	2	3	4	5	6	7	7+	Don't Know
12	**Which vegetables?**<br><br>How many servings of **carrot or pumpkin** or do you eat **in one week**?									
	0	1	2	3	4	5	6	7	7+	Don't Know
13	How many servings of **dark green leafy vegetables (silver beet, spinach)** do you eat **in one week?**									
	0	1	2	3	4	5	6	7	7+	Don't Know
14	How many servings of tomatoes **(fresh, canned, concentrated)** do you eat **in one week?**									
	0	1	2	3	4	5	6	7	7+	Don't Know

Figure S1. *Questionnaire for Veggie Meter® Foods*

Multiple Pass 24h Dietary Recall
Quick List

**Time**	**Food Item**

Figure S2. *The Modified 24-hour MPR First Pass*

Stage Two- Detailed List
Multiple Pass 24h Dietary Recall

Location	Time	Description<br>Product name<br>Home-made or retail<br>Cooking method	Amount Served	Brand	Leftover	To be completed by nutritionist	
						Food Group	Weight

Figure S3. *The Modified 24-hour MPR Second Pass*

Date________________________________ DAY 1

Time and place food was eaten	Complete description of food (food and beverage name, brand, variety, preparation method)	Amount consumed

Figure S4. *Weighed Food Diary*

Characteristic	Boys	Girls	Total
n	20	12	32
Age (years)	11 ± 1.293	11 ± 0.9	11 ± 1.2
Height (cm)	153 ± 13	153 ± 10	153 ± 12
Weight (kg)	43 ± 12	45 ± 12	44 ± 12
Body Mass Index (kg/m^2)	18 ± 2.9	19 ± 3.4	18 ± 3.1
Day one + two VM® Score	280 ± 66	286 ± 107	287 ± 82
Day one + two VM® Score (µmol/L)	1.6 ± 0.4	1.6 ± 0.6	1.6 ± 0.5
Ethnicity			
Māori	2	1	3
Tongan	2	0	2
European	16	10	26
Arab	0	1	1

Data are mean ± standard deviation.

VM®, Veggie Meter spectrophotometer.

Figure S5. *Participant characteristics*

Nutrient Intake	Total Weekday					Boys					Girls				
	Mean	Median	SD	Min	Max	Mean	Median	SD	Min	Max	Mean	Median	SD	Min	Max
Energy (kcal)	2143	2062	513.6	1093	3726	2199	2024	457.9	1785	3726	2050	2219	604.9	1093	3018
Carbohydrate (g)	253.2	245.7	60.04	138.9	415.7	257.5	247.5	52.36	188.1	415.7	246.1	221.1	73.05	138.9	396.5
Protein (g)	87.46	86.82	31.22	32.65	171.0	91.05	85.42	28.80	50.69	171.0	81.47	88.08	35.36	32.65	139.3
Total fat (g)	81.21	75.54	25.85	30.36	148.5	83.28	76.91	23.60	50.67	148.5	77.76	72.45	30.01	30.36	129.8
Saturated fat (g)	33.35	32	11.67	4.68	57.97	33.39	32	8.590	15.62	48.74	33.30	31.84	16.01	4.68	57.97
Dietary fibre (g)	24.87	24.71	9.837	9.65	54.95	27.28	27.77	10.38	13.6	54.95	20.85	23.31	7.651	9.65	31.08
Carotenoids (µg)	3862	1855	4539	186.3	17179	4033	1920	4519	234.9	17179	3577	1855	4758	186.3	13553
Sodium (mg)	2955	2825	1205	1145	5733	2938	2891	1113	1263	5733	2984	2825	1397	1145	5270
Calcium (mg)	931.7	922.4	492.7	134.4	1842	914.9	857.8	498.4	250.6	1654	959.8	952.6	503.5	134.4	1842
Iron (mg)	12.05	11.33	6.484	3.3	35.2	12.03	11.11	6.732	3.3	35.2	12.08	11.60	6.341	3.47	24.1
Fruit (serves)	1.573	1.225	1.583	0	7.47	1.808	1.425	1.802	0	7.47	1.183	0.81	1.089	0	3.17
Vegetable (serves)	2.351	1.755	1.944	0	8.61	2.332	2.07	2.031	0	8.61	2.383	1.755	1.879	0	5.78
Total F/V (serves)	3.924	3.47	2.527	0.07	11.08	4.139	3.965	2.818	0.07	11.08	3.565	2.38	2.014	1.26	6.72

Figure S6. *Weekday Modified 24-hour MPR Food Intake*

Nutrient Intake	Total Weekday					Boys					Girls				
	Mean	Median	SD	Min	Max	Mean	Median	SD	Min	Max	Mean	Median	SD	Min	Max
Energy (kcal)	2124	1974	614.5	749.3	3443	2188	2008	566.7	1535	3443	2017	1897	699.8	749.3	3074
Carbohydrate (g)	245.4	245.1	84.40	96.39	445.8	258.2	251.13	81.46	133.9	445.8	224.1	220.0	88.43	96.39	412
Protein (g)	82.17	82.15	30.70	24.09	164.9	90.27	86.05	30.83	33.13	164.9	68.68	75.21	26.41	24.09	113.7
Total fat (g)	85.08	79.8	30.02	25.65	170.9	81.45	74.11	22.91	27.63	120.0	91.14	89.10	39.60	25.65	170.9
Saturated fat (g)	34.40	34.23	12.71	5.29	62.67	33.86	32.85	10.72	10.17	59.96	35.29	35.69	15.99	5.29	62.67
Dietary fibre (g)	21.98	19.41	8.698	11.5	47.28	23.31	20.61	9.245	12.91	47.28	19.77	16.45	7.553	11.5	32.86
Carotenoids (µg)	1815	1093	1803	143.7	7020	2058	1305	1782	143.7	6542	1409	979.1	1841	157.0	7020
Sodium (mg)	2471	2313	817.9	1149	4138	2501	2528	757.2	1260	4138	2421	2245	943.7	1149	4134

Calcium (mg)	713.8	583.4	399.4	68.83	1552	773.2	710.5	424.1	154.5	1552	614.8	580.1	348.9	68.83	1248
Iron (mg)	11.23	9.885	5.561	1.74	28.06	11.83	10.46	5.588	3.63	28.06	10.24	9.545	5.612	1.74	21.06
Fruit (serves)	1.056	0.87	1.039	0	4.24	1.23	1.07	1.147	0	4.24	0.765	0.86	0.790	0	2.49
Vegetable (serves)	1.65	1.38	1.577	0	6.12	1.889	1.435	1.452	0	4.64	1.273	0.51	1.763	0	6.12
Total F/V (serves)	2.714	2.66	1.868	0	7.25	3.119	3.225	1.694	0	5.73	2.038	1.565	2.022	0	7.25

Figure S7. *Weekend Modified 24-hour MPR Food Intake*

Nutrient Intake	Total Weekday and Weekend Recall					Boys					Girls				
	Mean	Median	SD	Min	Max	Mean	Median	SD	Min	Max	Mean	Median	SD	Min	Max
Energy (kcal)	2133	2024	561.9	749.3	3726	2193	2024	508.6	1535	3726	2033	2086	640.1	749.3	3074
Carbohydrate (g)	249.3	245.7	72.76	96.39	445.8	257.9	249.8	67.59	133.9	445.8	235.1	221.1	80.11	96.39	412
Protein (g)	84.82	83.91	30.83	24.09	171.0	90.66	85.42	29.45	33.13	171.0	75.08	78.88	31.21	24.09	139.3
Total fat (g)	83.15	76.91	27.86	25.65	170.9	82.37	75.54	22.98	27.63	148.5	84.45	83.24	35.04	25.65	170.9
Saturated fat (g)	33.88	32.85	12.12	4.68	62.67	33.62	32.28	9.589	10.17	59.96	34.294	33.75	15.68	4.68	62.67
Dietary fibre (g)	23.43	23.27	9.325	9.65	54.95	25.30	23.73	9.906	12.91	54.95	20.31	19.82	7.455	9.65	32.86
Carotenoids	2838,	1426	3578	143.7	17179	3045	1646	3535	143.7	17179	2493	1037	3698	157	13553
Sodium (mg)	2713	2555	1050	1145	5733	2719	2596	965.4	1260	5733	2703	2383	1201	1145	5270
Calcium (mg)	822.7	778.5	458.2	68.83	1842	844.0,	811.8	462.4	154.5	1654	787.3	656	458.9	68.83	1842
Iron (mg)	11.64	10.65	6.006	1.74	35.2	11.93	10.89	6.108	3.3	35.2	11.16	10.1	5.931	1.74	24.1
Fruit (serves)	1.314	1.02	1.354	0	7.47	1.519	1.36	1.519	0	7.47	0.974	0.86	0.954	0	3.17
Vegetable (serves)	2.004	1.45	1.791	0	8.61	2.110	1.51	1.757	0	8.61	1.828	1.375	1.870	0	6.12
Total F/V (serves)	3.319	2.935	2.287	0	11.08	3.629	3.595	2.352	0	11.08	2.802	2.26,	2.122	0	7.25

Figure S8. *Average of Weekday and Weekend Modified 24-hour MPR Food Intake*

Nutrient Intake	Total Weekday					Boys					Girls				
	Mean	Median	SD	Min	Max	Mean	Median	SD	Min	Max	Mean	Median	SD	Min	Max
Energy (kcal)	2093	2128	572.9	917.3	3286	2205	2150	547.1	1275	3286	1908	2046	589.6	917.3	2746
Carbohydrate (g)	246.9	239.6	74.16	88.43	412.7	254.9	239.6	68.23	159.2	412.7	233.4	226.2	84.50	88.43	346.9
Protein (g)	79.58	78.67	29.96	31.17	184.1	86.63	79.15	29.91	44.36	184.1	67.83	64.80	27.26	31.17	123.1
Total fat (g)	82.30	80.44	26.31	44.2	153.5	87.65	88.33	27.51	44.62	153.5	73.39	69.47	22.47	44.2	117.4
Saturated fat (g)	33.60	31.27	12.22	11.99	61.28	35.85	34.63	11.51	17.32	61.28	29.84	27.99	12.95	11.99	54.16
Dietary fibre (g)	26.10	23.57	10.68	5.57	47.29	28.72	26.22	10.32	11.21	47.29	21.72	20.29	10.20	5.57	40.18
Carotenoids (µg)	3411	2181	3402	186.1	14199	3926	2616	3743	191.5	14199	2554	1743	2670	186.1	8035
Sodium (mg)	2689	2316	1042	1006	5693	2874	2555	1091	1682	5693	2381	2189	914.9	1006	4070
Calcium (mg)	1086	930.0	530.2	114.7	2564	1157	935.7	570.1	542.8	2564	967.8	858.1	454.3	114.7	1923
Iron (mg)	13.03	12.68	4.963	3.19	24.94	13.67	12.68	4.485	7.74	23.13	11.95	11.53	5.715	3.19	24.94
Fruit (serves)	1.167	0.77	1.161	0	4.98	1.347	1.04	1.336	0	4.98	0.868	0.66	0.751	0	2.96
Vegetable (serves)	2.271	1.71	1.851	0	8.76	2.338	1.915	2.020	0	8.76	2.16	1.41	1.610	0.5	5.24
Total F/V (serves)	3.438	2.9	2.386	0.38	11.23	3.684	3.165	2.535	0.38	11.23	3.028	2.205	2.156	0.5	8.2

Figure S9. *Weekday Weighed Food Diary Intake*

Nutrient Intake	Total Weekday					Boys					Girls				
	Mean	Median	SD	Min	Max	Mean	Median	SD	Min	Max	Mean	Median	SD	Min	Max
Energy (kcal)	2191	2083	574.3	1101	3257	2377	2271	551.4	1527	3257	1882	1939	486.9	1101	2697
Carbohydrate (g)	253.5	256.0	64.70	151.9	411.8	267.9	279.2	68.98	151.9	411.8	229.4	226.6	50.61	160.2	304.8
Protein (g)	86.38	79.61	38.80	24.99	214.8	97.42	84.54	40.33	36.72	214.8	67.98	59.20	28.99	24.99	115.0
Total fat (g)	87.53	81.65	30.54	29.44	158.0	96.16	88.17	29.82	46.42	158.0	73.14	74.38	27.03	29.44	115.1
Saturated fat (g)	35.31	34.18	13.28	15.1	68.84	38.96	37.66	14.02	20.5	68.84	29.22	30.59	9.638	15.1	43
Dietary fibre (g)	24.88	22.77	9.766	9.53	62.06	27.27	23.79	10.92	16.83	62.06	20.91	21.1	5.935	9.53	28.28
Carotenoids (µg)	1589	911.1	1432	261.8	5673	1706	1262	1288	268.7	5596	1394	702.7	1687	261.8	5673
Sodium (mg)	3026	2723	1169	1080	6107	3297	3191	1154	1796	6107	2574	2504	1093	1080	5010

Calcium (mg)	951.8	800.0	453.6	310.9	2460	1085	919.8	504.2	489.3	2460	730.4	722.7	234.7	310.9	1118
Iron (mg)	11.94	10.54	4.511	2.29	23.1	13.21	11.12	4.656	5.69	23.1	9.832	9.995	3.484	2.29	16.08
Fruit (serves)	1.344	1.12	1.041	0	3.46	1.519	1.21	1.018	0	3.43	1.052	0.775	1.055	0	3.46
Vegetable (serves)	1.473	1.32	1.355	0	5.51	1.644	1.505	1.140	0	4.85	1.189	0.365	1.670	0	5.51
Total F/V (serves)	2.817	2.5	1.767	0.3	7.8	3.163	3.505	1.738	0.73	7.8	2.241	2.225	1.733	0.3	6.64

Figure S10. *Weekend Weighed Food Diary Intake*

Nutrient Intake	Total Weekday and Weekend Weighed					Boys					Girls				
	Mean	Median	SD	Min	Max	Mean	Median	SD	Min	Max	Mean	Median	SD	Min	Max
Energy (kcal)	2142	2116	571.2	917.3	3286	2291	2219	549.2	1275	3286	1895	1962	529.0	917.3	2746
Carbohydrate (g)	250.2	246.9	69.11	88.43	412.7	261.4	262.7	68.04	151.9	412.7	231.4	226.6	68.15	88.43	346.9
Protein (g)	82.98	79.06	34.55	24.99	214.8	92.02	80.46	35.47	36.72	214.8	67.90	59.87	27.52	24.99	123.1
Total fat (g)	84.91	81.45	28.40	29.44	158.0	91.91	88.33	28.64	44.62	158.0	73.26	73.20	24.31	29.44	117.4
Saturated fat (g)	34.45	31.97	12.69	11.99	68.84	37.41	35.17	12.76	17.32	68.84	29.53	29.04	11.17	11.99	54.16
Dietary fibre (g)	25.49	22.92	10.17	5.57	62.06	28.00	25.35	10.51	11.21	62.06	21.31	20.29	8.174	5.57	40.18
Carotenoids (µg)	2500	1306	2747	186.1	14199	2816	2013	2983	191.5	14199	1974	842.8	2263	186.1	8035
Sodium (mg)	2857	2622	1112	1006	6107	3086	2852	1129	1682	6107	2477	2347	990.9	1006	5010
Calcium (mg)	1019	885.3	494.1	114.7	2564	1121	935.7	532.5	489.3	2564	849.1	803.7	373.9	114.7	1923
Iron (mg)	12.48	11.54	4.736	2.29	24.94	13.44	12.37	4.518	5.69	23.13	10.89	10.19	4.754	2.29	24.94
Fruit (serves)	1.255	0.99	1.098	0	4.98	1.433	1.135	1.175	0	4.98	0.96	0.66	0.901	0	3.46
Vegetable (serves)	1.872	1.44	1.659	0	8.76	1.991	1.625	1.657	0	8.76	1.675	1.16	1.679	0	5.51
Total F/V (serves)	3.128	2.76	2.106	0.3	11.23	3.424	3.255	2.161	0.38	11.23	2.635	2.225	1.955	0.3	8.2

Figure S11. *Average* of *Weekday and Weekend Weighed Food Diary Intake*
